# Corrigendum

**DOI:** 10.1002/rcr2.557

**Published:** 2020-04-06

The authors would like to draw the reader's attention to some errors in the following article:

Frankel, A, Ferris, R, Hodgkinson, P, Ellender, CM. (2020) Cholecystectomy under thoracic epidural anaesthesia for patient with severe intra‐pulmonary shunt. *Respirology Case Reports*, 8(3), e00543. https://doi.org/10.1002/rcr2.543

The affiliations for Rebekah Ferris and Agustina Frankel and Peter Hodgkinson are as follows:

Drs Rebekah Ferris and Agustina Frankel: Princess Alexandra Hospital, Department of Anaesthesia, Woolloongabba, QLD, Australia.

Dr. Peter Hodgkinson: Princess Alexandra Hospital, Department of Hepatobilliary and Transplant Surgery, Woolloongabba, QLD, Australia.

In the Introduction:

“alveolar partial pressure of oxygen (PaO2)” should read “arterial partial pressure of oxygen (PaO2)” in the text.

In the legend of Figure 2:

‐ “PaCO2, alveolar partial pressure of carbon dioxide” should read “PaCO2, arterial partial pressure of carbon dioxide”.

‐ “PaO2, alveolar partial pressure of oxygen” should read “PaO2, arterial partial pressure of oxygen”.

‐ “Pc'O2, estimated from calculated PaO2 (ideal alveolar air equation)” should read “Pc'O2, estimated from calculated PAO2 (ideal alveolar air equation)”.

The authors apologize for this error and any confusion it may have caused.

